# Familial Hypercholesterolemia: A Literature Review of the Pathophysiology and Current and Novel Treatments

**DOI:** 10.7759/cureus.49121

**Published:** 2023-11-20

**Authors:** Yasha N Suryawanshi, Rupesh A Warbhe

**Affiliations:** 1 Department of Physiology, Jawaharlal Nehru Medical College, Datta Meghe Institute of Higher Education and Research, Wardha, IND; 2 Department of Pharmacology, Jawaharlal Nehru Medical College, Datta Meghe Institute of Higher Education and Research, Wardha, IND

**Keywords:** heterozygous familial hypercholesterolemia, homozygous familial hypercholesterolemia, low density lipoprotein-cholesterol, novel therapeutics, current therapeutics, pathophysiology of familial hypercholesterolemia, familial hypercholestrolemia

## Abstract

Familial hypercholesterolemia (FH) is a genetically transmitted disorder. It shows an autosomal dominant mode of inheritance. It is a metabolic disorder. Mutation in chromosome 19 leads to this disorder. Chromosome 19 codes data for low-density lipoprotein (LDL) receptor (LDLR). LDLR either reduces increased LDL levels from the circulation or maintains a normal level of LDL. It precipitates the risk of cardiovascular disease at an early age. Characteristic features of FH are elevated levels of LDL in the blood because of sudden changes in LDLR, which causes a decrease in the clearance of LDL from the blood. Plaque gets deposited in the lumen of the arteries, called atherosclerosis, which occurs at an early young age. If both genes are affected then it is homozygous FH (HoFH); such a case is very rare. When a single gene is affected then that condition is known as heterozygous FH (HeFH). HoFH comes up with more severe cardiac disease than HeFH at an early age. The major cause of FH is a mutation in the LDLR gene while other causes include mutation in various genes like apolipoprotein B (apo B), proprotein convertase subtilisin/kexin type 9 (PCSK9), LDLR adaptor protein 1 (LDLRAP 1). In order to prevent cardiovascular crises due to FH, it must be diagnosed early and treated effectively. With increasing research and advances in medical sciences, many kinds of current and novel therapies are emerging that aim to reduce the level of LDL in blood.

## Introduction and background

Familial hypercholesterolemia (FH) is a gene-related metabolic disorder. This autosomal dominant disease is defined as significantly raised low-density lipoprotein (LDL) cholesterol levels since birth, leading to the increased risk of cardiac manifestation at a young age, which can be fatal [1]. In general, the occurrence of FH in unselected general populations has been assessed to be one in 200-250 in heterozygous FH (HeFH) and one in 160,000-300,000 in homozygous FH (HoFH). This indicates most inherited hereditary disorders in human beings may be FH [1]. The mutated gene responsible for FH is located on chromosome 19. This chromosome 19 carries information regarding a protein called LDL receptor (LDLR), and it removes LDL cholesterol from circulation. One in 500 people has an affected gene that causes FH. If only one allele is affected, then it is known as HeFH, and if a person inherits the gene alteration from both parents, it is called HoFH. HoFH is a significantly major form of hypercholesterolemia, with the occurrence of cardiovascular disease (CVD) and demise at an early age [2].

FH exposes people to high LDL cholesterol levels for the rest of their lives. It is not uncommon, but it is often underdiagnosed. Though there are treatments for FH, it is not treated properly. Preliminary detection and management reduce an increased premature CVD resulting from FH [3]. This disorder is detected by clinical characteristics, ancestral history, and raised LDL levels, which have currently been supported by gene-based therapeutics. Early identification and treatment are essential since the degree and scope of contact with LDL cholesterol levels dictate atherosclerotic stress. Though new drugs, LDL apheresis, and other investigational treatments may be helpful for some hard-to-treat subsets of FH, statins are most commonly prescribed and are important treatments for these patients. Collectively, these new emerging therapies have significantly enhanced the outcome of FH, particularly in heterozygous individuals [4].

Many FH patients require multiple medications to achieve optimal LDL cholesterol lowering. According to LDL levels and how they respond to treatment, patients might need multiple medications. Ezetimibe, bile-acid sequestrants, and niacin are among the drugs that can be combined with statins to reduce LDL cholesterol [3]. LDL apheresis, which is nonsurgical therapy, is a significant management choice for both types of FH sufferers who fail to attain their treatment objectives despite receiving optimal tolerated medical therapy. Monoclonal antibodies to PCSK9 are the most highly ensuring recent treatment for FH [3]. Nutritional pharmacological substances that are naturally lipid-controlling agents are advised in conjunction with the treatment of hypercholesterolemia. It affects lipid metabolism, enhancing the consequences of various lipid-lowering treatments [5]. If left untreated, FH enhances the possibility of CVD. Maintained elevated cholesterol levels promote oxidation of lipoproteins and intrusion through the endothelial hurdle, and hastening atherosclerotic plaque progression [5].

## Review

Methodology

**Figure 1:**
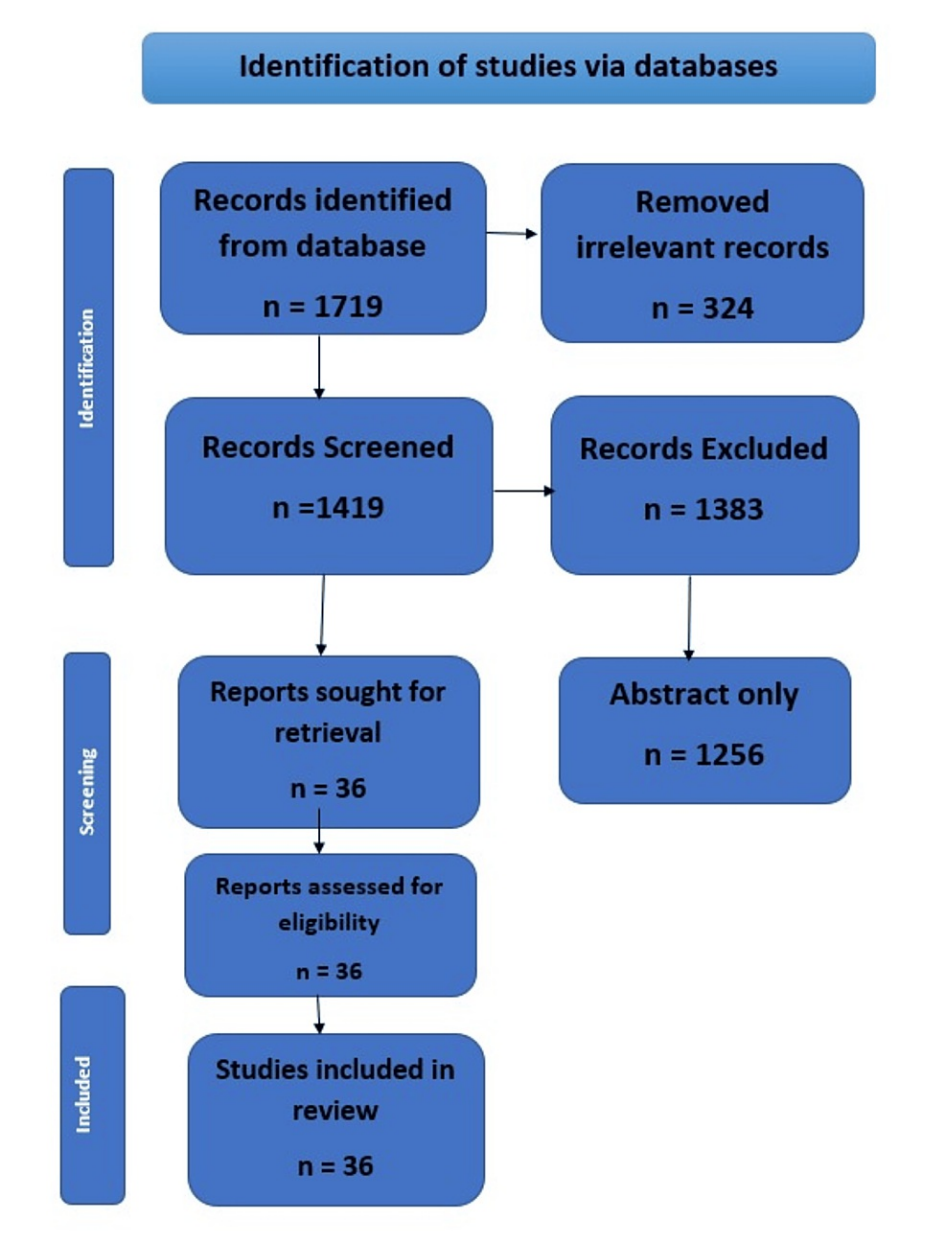
PRISMA flowchart showing the selection process of articles reviewed PRISMA: Preferred Reporting Items for Systematic Reviews and Meta-Analysis

We performed a systematic search through PubMed Central in July 2023 using keywords and Medical Subject Headings (MeSH) such as “familial hypercholesterolemia” and “pathophysiology” and “pharmacological therapies” (((familial hypercholesterolemia [Title/Abstract]) OR (novel therapies[Title/Abstract])) OR (pathophysiology*[Title/Abstract])) OR (“Heterozygous familial hypercholesterolemia” [MeSH Terms]) AND ((“Homozygous familial hypercholesterolemia” [Title/Abstract]) OR (Current therapies to treat familial hypercholesterolemia [Title/Abstract])) OR (“Novel therapies to treat familial hypercholesterolemia” [MeSH Terms]). We additionally searched for key references from the bibliographies of the relevant studies. Figure 1 shows the Preferred Reporting Items for Systematic Reviews and Meta-Analyses (PRISMA) flowchart that was followed.

Pathophysiology of FH

FH occurs widely because of mutation in the following genes: LDLR gene, apolipoprotein B (Apo B), proprotein convertase subtilisin/kexin type 9 (PCSK 9), LDLR adaptor protein 1 (LDLRAP1), and other unidentified genes [6]. In the body, LDL normally circulates for some time then eventually the Apo B portion in LDL attaches to LDLR on the hepatocytes leading to the uptake of LDL and its digestion [7]. LDL is cleared from the circulatory system as a result of this process. The 3-hydroxy-3-methylglutaryl-coenzyme A reductase (HMG-CoA reductase) enzyme synthesizes cholesterol, this enzyme is inhibited resulting in the lowering of cholesterol levels [8]. LDL receptor is absent or reduced in FH which leads to the presence of LDL in circulation for 4.5 days causing elevated LDL levels in blood [9]. Frequent genetic abnormalities in FH are LDLR loss of functional mutations (among 250 single individuals affected), Apo B functional loss, PCSK9-acquired functional alterations, and LDLRAP alterations [6].

LDL Receptor Gene

Chromosome 19 has a p arm containing the LDL gene. In about 40% of cases by the age of 50, only one altered gene as in HeFH leads to heart disorder. Having two altered genes as in HoFH causes accelerated heart disorders during the young years of life with severe complications. The action of LDLR among homozygotes is below 2%, but in the case of HeFH having flawed LDL activity with its receptor, it is 2-25%, on the basis of the type of the mutation [10]. There are five main classes that depict types of LDLR mutations [11]. There is no synthesis of LDLR in class 1 while there is difficulty in the transferring of LDLR from the endoplasmic reticulum to the Golgi apparatus in class 2. In class 3, apo B is defective, which leads to improper binding between LDLR and hepatocyte cells. In class 4, there is a defect in cluster clathrin-coated pits receptor leading to improper endocytosis. In class 5, the recycling of LDLR is hampered.

Apo B

Lipoprotein particles consist of a protein part called apolipoprotein B. The gene that codes it, is 46.2 kb in length, located on chromosome 2 (2p24-p23). FH is generally linked with the mutation in R3500Q, leading to a replacement of glutamine for arginine at 3500 places. Binding is improper as there is a structural change in the protein that attaches to the LDLR. The higher the amount of abnormal copies of LDLR, the higher the cholesterol levels [12].

PCSK9

Familial cholesterolemia also occurs due to mutation in proprotein convertase subtilisin/kexin type 9. Chromosome one carries this gene [1].

LDLRAP 1

LDLRAP1, also known as ARH (adaptor-related protein homolog), was identified in FH [13]. Protein mutations typically result in the production of less protein. Its true mechanism of action is unknown; it may be involved in the interaction of clathrin-coated pits with LDLRs [14]. Table 1 depicts genes undergoing mutations in FH, their mechanism of action, and the mutated effects of those genes [3].

**Table 1:** Genes involved in familial hypercholesterolemia, mutation in those genes, their mechanism of action, mutated effect of the gene LDLR: low density lipoprotein receptor; LDL: low density lipoprotein; LDLRAP1: low-density lipoprotein receptor adaptor protein 1; Apo B: apolipoprotein B; PCSK9: proprotein convertase subtilisin/Kexin type 9

Gene	Mutation	Mechanism of action	Mutated effect
LDLR	LDLR	LDLR mediates the endocytosis of cholesterol-rich LDL and thus maintains the plasma level of LDL	LDLR may be absent or decreased leading to increased plasma levels of LDL
LDLRAP1	LDLR adaptor protein	Protein used in clathrin-mediated internalization of LDLR	Mutations lead to LDLR malfunction
Apo B	Apo B	It is required for LDL to bind with LDLR	Impairment in binding with LDLR
PCSK9	PCSK9 gain of function	PCSK9 promotes the degradation of LDLR	Elevated PCSK9 levels damage LDLRs

Current treatment therapies for FH

Statins

Stains are the most important drug used in the medication of FH. It is often used with other lipid-lowering drugs. Statins are routinely used as therapeutic drugs reducing raised cholesterol levels. It acts mainly by inhibiting the HMG-CoA (hydroxy methyl glutaryl-coenzyme A) reductase enzyme, this enzyme regulates the overall metabolism of cholesterol in the body [15]. Statin blocks the active site in this enzyme. Due to the inactivation of the site, HMG-CoA is not converted into mevalonic acid as it blocks access to the substrate. Hence cholesterol formation is reduced leading to increased expression of microsomal HMG-CoA and LDL receptor expression on the hepatocyte. LDL cholesterol clearance is increased, thus lowering LDL cholesterol levels by 20-55% in circulation. Statins not only reduce LDL but also have other advantages like it decreases the chances of having dementia, reducing inflammation, improving endothelial function, and making atheromatic plaque stable [16]. Statin therapy leads to enhanced production of LDLR [17]. Some patients in spite of being sensitive to statins may respond less to statins than other FH patients as statins may also function by reducing the formation of very low-density lipoprotein. Reduction in LDL cholesterol observed in these people is less as compared to those who have hypercholesterolemia, which is acquired [18].

PCSK9 Inhibitor

PCSK9 is mostly found in the liver and is important for LDLR production as it binds to hepatocytes on the protein's surface and directs its destruction. As a result, the gene PCSK9 is normal but it is expressed in an abnormal form; this is called a gain of function mutation, which leads to the development of FH, and high levels of PCSK9 are linked to hypercholesterolemia [19]. A number of PCSK9 inhibitors have been created, including monoclonal antibodies (evolocumab and alirocumab) that attach to PCSK9 in circulation, thus preventing it from collaborating with surface LDLR as well as small interfering ribonucleic acid (RNA) that block PCSK9 production within cells. Numerous increased cholesterol populations, including those with FH, have been investigated using monoclonal antibodies [20].

Ezetimibe

It limits the action of a transporter called Niemann-Pick C1 like 1 (NPC1L1). It inhibits the uptake of cholesterol through diet and cholesterol secretion in bile. Reduced uptake of the liver increases the low-density lipoprotein receptors these receptors utilize LDL cholesterol, hence reducing LDL cholesterol levels. Statins along with ezetimibe reduce LDL cholesterol levels significantly [21].

Mipomersen

It binds to the human ApoB mRNA's coding region and causes its breakdown. This reduces atherogenic Apo B. This Apo B contains lipoproteins like LDL and very low-density lipoprotein. It inhibits apo B lipoprotein as it is an antisense oligonucleotide. The expression of LDLR is not necessary for lowering LDL cholesterol levels caused by mipomersen [22]. Since mipomersen is not a strong CYP isoenzyme inhibitor it cannot be metabolized by the cytochrome system. Hence, reactions among mipomersen and statins or ezetimibe are not anticipated. It has been created as an additional therapy for FH patients, particularly for those with HoFH [23].

Lomitapide

The function of the protein, microsomal triglyceride protein (MTP) is the production of LDL and chylomicrons in the hepatocytes and enterocytes [22]. MTP inhibitor is a potential pharmaceutical agent for the management of raised cholesterol levels above normal value. Due to this, lomitapide, an MTP inhibitor, was created and is now authorized for the management of HoFH [24].

Lipoprotein Apheresis

It is a procedure in which lipoproteins from circulation are removed and hence used in the management of FH. Lipoprotein apheresis is done when drugs are ineffective in decreasing LDL. It can be done in addition to lipid-lowering drugs. It is recommended for HoFH and critical forms of HeFH, patients with HeFH not responding to statin therapy, or for patients intolerant to therapy [25]. 

Bile Acid Sequestrants

They are used in the digestive system by binding to bile constituents and it is a type of resin. They interfere with the bile component's reabsorption from the gut by interacting with them and blocking bile acid's enterohepatic circulation. Bile acid sequestrants have properties of polymeric agents. They function like resins and exchange like minerals. Bile acid sequestrants combine with bile acid for the transfer of chloride ions. Because of this binding, bile acids are cleared from the enterohepatic circulation. For restoring bile acid levels, the liver starts to synthesize bile acids from cholesterol, hence cholesterol from blood is utilized, which reduces the levels of LDL [26]. Table 2 depicts various current drug therapies utilized in the treatment of FH [3].

**Table 2:** Current drug therapies used to treat familial hypercholesterolemia HMG-CoA: 3-hydroxy-3-methyl-glutaryl-coenzyme A; CYP3A4: cytochrome P450 3A4; PCSK9: proprotein convertase subtilisin/Kexin type 9; LDL: low density lipoprotein

Drug	Mechanism of action	Pharmacologic effect of this drug on various systems and safety issues
Statins: rosuvastatin, atorvastatin, lovastatin, pravastatin	Competitively inhibiting the active site of HMG-CoA reductase, and restricts substrate access	Low dose in deranged renal function, Asian continent, and old patients. Minimal renal excretion CYP3A4 substrate
PCSK9 Inhibitor: alirocumab, evolocumab	Alirocumab, evolocumab are antibodies that binds in plasma to PCSK9, promoting degradation of the PCSK9	Injection-site reactions, generally mild, nasopharyngitis
Ezetimibe	Ezetimibe remains on surface of brush border of small intestine, preventing cholesterol absorption	Abdominal pain, diarrhea, myalgia
Mipomersen	It binds to mRNA blocking apolipoprotein B 100 synthesis. This leads to decrease LDL synthesis	Raised hepatic fat. thrombophlebitis, weakness, nausea
Lomitapide	Lomitapide lowers LDL cholesterol levels by a mechanism unrelated to LDLR	Elevated hepatic fat, diarrhea, gastrointestinal disturbances.
Bile acid sequestrants colesevelam colestipol cholestyramine	The body utilizes cholesterol to generate bile acids, so there is reduction in LDL cholesterol blood levels	It should ideally consumed with food. It has gastrointestinal side effects. It also interacts with absorption of other drugs like Interference with the absorption of other drugs.

Novel treatment therapies for FH

Inclisiran

It is a synthesized small interfering RNA (siRNA) with long action. Inclisiran is useful for lowering highly raised LDL levels in blood, it is also beneficial in the treatment of other CVDs. Inclisiran is very successful in lowering bad cholesterol levels. It binds to specific liver receptors and prevents the production of PCSK9 by doing so. This drug is sold under the trade name Leqvio [27]. The siRNA functions as a proprotein convertase inhibitor to prevent the translation of the protein PCSK9 [15]. Inclisiran use was approved in the European Union. In August 2021, NICE approved it for use by the UK's National Health Service. In the United States, it was approved for medical use in December 2021. The medication is rated as the best in its class by the American Food and Drug Administration.

Angiopoietin-Like Protein 3 (ANGPTL3) Inhibitors

It is advised for treatment of FH which is resistant to statin therapy. ANGPTL3 blockers have come to light as a capable drug addition to the arsenal of cholesterol-lowering treatments. ANGPTL3 is a glycoprotein involved in metabolism and it is secreted by hepatic cells. It reduces the levels of triglycerides, LDL cholesterol, and total cholesterol by inhibiting endothelial lipase and lipoprotein lipase synthesis. ANGPTL3 gene shows loss of function mutation means it’s a genetic lesion that results either in a decrease in its genetic activity or inhibition of synthesis [28]. After thorough clinical trials, this drug was declared effective and safe for use and it was approved by the United States Food and Drug Administration (FDA). ANGPTL3 is inhibited when a monoclonal antibody targets and binds with it. For patients of HoFH aged 12 years or more, this drug is used in addition to other lipid-lowering drugs [29].

Bempedoic Acid

This drug is marketed under the trade name Nexletol. The hepatic enzyme produces cholesterol. Cholesterol production is blocked by bempedoic acid [30]. This enzyme is called ATP citrate lyase. The United States approved the therapeutic usage of bempedoic acid in February 2020, whereas it was approved for therapeutic usage in Europe in April 2020 [31]. FDA declared bempedoic acid as a first-class drug to use in treatment therapy. European Union suggests bempedoic acid for adults with primary hypercholesterolemia, dyslipidemia, and FH. If patients are unable to reduce high levels of LDL or unable to maintain normal LDL cholesterol levels in spite of the maximum tolerable dose of statin, in such cases bempedoic acid can be added along with statins and other lipid-lowering drugs [32].

Gemcabene

It is a comparatively new drug. It quickens the clearance of very low-density lipoprotein. This drug reduces hepatic apolipoprotein C-III (Apo C-III) and it lowers LDL. Gemcabene enhances HDL (high-density lipoprotein) cholesterol and reduces LDL cholesterol, and triglycerides levels in the blood [33].

RNA Targeted Therapeutics

This therapy causes defects in the mechanism of the translation process and it affects the nucleic acids which code the proteins and try to regulate their action in abnormal ways [34]. Some examples of RNA-targeted therapies are upregulation of mRNA, modulation of RNA splicing oligonucleotide, miRNA mimics, and inhibitors. They don't integrate into the host DNA, are synthetic and tiny, and only have a short lifespan and activity [35].

DNA Targeted Therapeutics

DNA-targeted therapy typically aims to have the opposite impact as compared to RNA-targeted therapeutics. This therapy is practiced for selectively silencing and disrupting genes to show their genetic appearance. To make a mutant gene functional again, this method includes inserting functional gene copies [36].

Table 3 presents an analysis of the traits and features of the articles included in the review

**Table 3:** Characteristics of the study included in the article LDL: low density lipoprotein; HMG CoA: β-hydroxy β-methylglutaryl-CoA; PCSK9: proprotein convertase subtilisin/Kexin type 9; Apo B: apolipoprotein B; mRNA: messenger ribonucleic acid; ATP: adenosine triphosphate; RNA: ribonucleic acid; DNA: deoxyribonucleic acid

Author	Year of publication	Findings
Tokgozoglu et al. [1]	2021	Findings include information regarding familial hypercholesterolemia, it is autosomal dominant genetic disease, it occurs due to increases low density lipoprotein levels and its types.
Bouhairie et al. [3]	2015	According to the findings familial hypercholesterolemia remains underdiagnosed and undertreated. If it is diagnosed early and treated then risk of cardiovascular disease is reduced.
Varghese et al. [4]	2014	Findings suggests various treatment therapies, drugs like statins, other lipid lowering drug are used in treatment of familial hypercholesterolemia.
Benito-Vicente et al. [5]	2018	Familial hypercholesterolemia is an autosomal genetic disorder because of a defect in lipid metabolism resulting in the early onset of cardiovascular disease.
Akioyamen et al. [6]	2017	Genes involved in familial hypercholesterolemia are low density lipoprotein receptor gene, apolipoprotein B, proprotein convertase subtilisin/kexin type 9, low density lipoprotein receptor adapter protein 1, other unidentified genes.
Brown et al. [8]	1974	It gives information about pathogenesis of familial hypercholesterolemia.
Durrington et al. [9]	2003	LDL receptors are reduced or absent in familial hypercholesterolemia .
Rador et al. [10]	2003	Genetic mutation leading to familial hypercholesterolemia are described in this finding.
Hobbs et al. [11]	1992	Low density lipoprotein receptor leads to uptake utilization and breakdown of low density lipoprotein.
Vega et al. [12]	1986	Information about apolipoprotein B and its mutation .
Zubieliene et al. [15]	2022	Early diagnosis and treatment of familial hypercholesterolemia, and about drugs likestatins, ezetimibe, bile acid sequestrants, niacin, pcsk9 inhibitors ,lompitamide, mipomersin, RNA based therapeutics.
Ward et al. [16]	2019	Findings about statin therapy it is first line of treatment in familial hypercholesterolemia , it acts by inhibiting HMG-CoA reductase enzyme.
Feher et al. [17]	1993	It gives information about homozygous familial hypercholesterolemia and cholesterol lipid lowering drug effect of statin.
Raal et al. [18]	2000	Atorvastatin reduces cholesterol by inhibiting its synthesis in homozygous familial hypercholesterolemia.
Abifadel et al. [19]	2003	PCSK9 inhibitor used in treatment of familial hypercholesterolemia.
Benn et al. [20]	2010	It gives information about genetic mutation in PCSK9 gene.
Cannon et al. [21]	2015	Management of familial hypercholesterolemia by using other lipid lowering drug along with statins. Ezetimibe drug inhibits absorption of cholesterol from diet as well as biliary cholesterol.
Crooke et al. [22]	2013	Mipomersin leads to destruction of ApoB mRNA by binding with it. This leads to decreased production of low density lipoprotein, very low density lipoprotein.
Geary et al. [23]	2015	Mipomersin drug used in treatment of familial hypercholesterolemia, which is used as antisense oligonucleotide and it acts by inhibiting apolipoprotein B.
Raal et al. [24]	2010	Effectiveness of mipomersin in treatment of homozygous familial hypercholesterolemia by reducing in concentration of low density lipoprotein.
Stefanutti et al. [25]	2017	Lipoprotein apheresis is a procedure in which certain lipoproteins are eliminated from blood. It is suggested for homozygous familial hypercholesterolemia and in critical case of heterozygous familial hypercholesterolemia.
Hashim et al. [26]	1965	Bile acid sequestrants as emerging novel therapy in treatment of familial hypercholesterolemia. Bile acids are removed from circulation and to restore their levels cholesterol is utilised this decrease blood cholesterol level.
Wright et al. [27]	2021	Information about inclisiran, its mechanism of action, use in treatment of familial hypercholesterolemia. It inhibits the production of PCSK 9.
Surma et al. [28]	2023	Angiopoietin-like protein 3 gene is mutated leading to raised cholesterol levels in blood.
Fukuhara et al. [29]	2020	Angiopoietin-like protein 3 gene used in management of familial hypercholesterolemia.
Lawrence et al. [30]	2021	Enzyme ATP citrate lyase is inhibited by bempedoic acid resulting in reduction of cholesterol.
Ballantyne et al. [32]	2022	Bempedoic acid effective drud in treatment of heterozygous familial hypercholesterolemia.
Besekar et al. [33]	2023	Findings suggests that gemcabene is effective anti-inflammatory drug and cholesterol lowering effect, progression of fibrosis.
Chen et al. [34]	2022	Emerging novel treatment for management of familial hypercholesterolemia.
McClorey et al. [35]	2015	Information about RNA targeted therapied for treatment of familial hypercholesterolemia.
Defesche et al. [36]	2017	Findings include information on DNA targeted therapy in treatment of familial hypercholesterolemia

## Conclusions

A genetically acquired condition, FH occurs due to mutated genes that code for certain proteins regulating the LDL metabolism, resulting in the early onset of CVD because of raised contact with LDL cholesterol since an early age. Despite being one of the most prevalent autosomal dominant hereditary genetic illnesses, it frequently goes underdiagnosed and untreated. To treat patients effectively, it's essential to comprehend the pathophysiology of the illness as well as the significance of cascade screening. However, various drug therapies have emerged and are currently in use to treat FH. Statins are the preferred drugs in the treatment of FH. Many people also need ezetimibe and/or PCSK9 inhibitors for effectively lowering LDL cholesterol. The major drug in managing both types of FH is statin. However, along with statins, some other drugs are also used in combination like ezetimibe, bile acid sequestrants, PCSK9 inhibitors, and others. When pharmaceutical treatment is insufficient, lipoprotein apheresis is performed. It is also required in both types of hypercholesterolemia. People suffering from HoFH can be advised mipomersen and lomitapide drugs. Future research on novel therapies to treat FH is constantly going on.
